# Association of Neutrophil-to-Lymphocyte Ratio With Early Diabetic Nephropathy in Patients With Type 2 Diabetes Mellitus

**DOI:** 10.7759/cureus.105087

**Published:** 2026-03-12

**Authors:** Geetika Gupta, Puneeta Gupta, Anil K Gupta, Sunny Babber

**Affiliations:** 1 Department of Physiology, Acharya Shri Chander College of Medical Sciences and Hospital, Jammu, IND; 2 Department of Medicine, Acharya Shri Chander College of Medical Sciences and Hospital, Jammu, IND; 3 Department of Community Medicine, Acharya Shri Chander College of Medical Sciences and Hospital, Jammu, IND

**Keywords:** albuminuria, end-stage renal disease, inflammatory markers, neutrophil–lymphocyte ratio, renal markers, type 2 diabetes mellitus

## Abstract

Background: Type 2 diabetes mellitus (T2DM) is a chronic condition associated with a range of complications, one of the most significant being diabetic nephropathy (DN). It is a leading cause of kidney failure, and its early detection is crucial for delaying disease progression. While traditional markers are commonly used, they often detect renal impairment only in more advanced stages. Since chronic inflammation is a key contributor to the development of diabetic complications, the neutrophil-to-lymphocyte ratio (NLR) may serve as a useful and cost-effective biomarker of early DN in patients with T2DM.

Objective: To determine whether elevated NLR levels are associated with early markers of renal dysfunction, and to assess the predictive value of NLR in identifying early-stage DN in type 2 diabetic patients.

Methods: A total of 126 T2DM patients satisfying the inclusion criteria were included in the study after taking informed consent. Subjects were divided into two groups based on the presence (66) and absence (60) of albuminuria. Fasting blood sugar, glycated hemoglobin, NLR, blood urea, serum creatinine, urinary albumin excretion, urinary albumin creatinine ratio, and estimated glomerular filtration rate were noted to assess renal function.

Results: A total of 126 patients diagnosed with type 2 diabetes were registered in our study. The mean NLR for patients without albuminuria was 1.96 ± 0.54, and for the DN group, it was 3.06 ± 0.98, which was significant (p < 0.001). Also, a strong positive correlation was seen between NLR and markers of early DN (r = 0.42, p = 0.001).

Conclusion: A significant correlation was found between NLR and markers of early DN in patients with T2DM. NLR may be considered a cost‑effective surrogate biomarker for the early detection of DN, enabling timely intervention strategies to mitigate progression of nephropathy in T2DM.

## Introduction

Type 2 diabetes mellitus (T2DM) ranks among the most common chronic conditions globally and causes severe microvascular and macrovascular complications, substantially increasing mortality risk in affected patients ​​[1,2]. The prevalence of T2DM is increasing rapidly due to economic development and lifestyle modifications. In 2021, T2DM affected around 10.5% of the world’s adult population between 20 and 79 years old, which translates to approximately 537 million people globally. Projections estimate this could exceed 1.31 billion by 2050, with comparable rates between men and women [3].

Diabetic nephropathy (DN) constitutes a frequent microangiopathic disorder in diabetes patients and is recognized as one of the foremost causes of end-stage renal disease (ESRD) [4]. Clinically, DN presents as progressive albuminuria, starting with microalbuminuria, advancing to macroalbuminuria, and eventually leading to ESRD [5]. In recent years, the role of inflammatory processes in T2DM development and associated metabolic complications has gained increasing recognition [6]. Several inflammatory markers, including interleukin-1 (IL-1), interleukin-6 (IL-6), tumor necrosis factor alpha (TNF-α), and other cytokines, have been associated with the development and progression of DN. However, routine clinical measurement of these markers is limited due to high costs and technical complexities [7]. Recently, the neutrophil-to-lymphocyte ratio (NLR) has become a promising inflammatory biomarker, providing a more comprehensive assessment of systemic inflammation than individual markers. It offers cost-effectiveness and ease of measurement [8].

Common diabetes risk factors, including obesity, physical inactivity, smoking, poor diet, psychological stress, and infections, activate the innate immune system, initiating chronic low-grade inflammation. This sustained inflammatory state, frequently marked by elevated leukocyte counts, contributes significantly to diabetic complication development [9] and is associated with altered inflammatory cytokine levels [10]. Pro-inflammatory cytokines promote insulin resistance via molecular pathways, including c-Jun N-terminal kinase (JNK) activation, IκB kinase-β/nuclear factor κB (IKKβ/NF-κB) signaling activation, and decreased peroxisome proliferator-activated receptor gamma (PPARγ) expression, a protein crucial for enhancing insulin sensitivity [11-13]. In T2DM patients, maintaining optimal glycemic control is strongly recommended based on epidemiological evidence to prevent or delay vascular complication onset and progression [14].

However, scientific literature examining the relationship between NLR values and DN remains limited. This research aims to assess NLR's potential as a predictor of inflammatory processes and DN in individuals with T2DM.

## Materials and methods

Study design and participant selection

This research was designed as a prospective, observational, non-interventional study after getting approval from the Institutional Independent Ethics Committee (Reference No.: ASCOMS/IEC/RP&T/2020/385; dated: 25/07/2020). The study was conducted from August 1, 2022, to January 31, 2024, at the Department of Physiology, Acharya Shri Chander College of Medical Sciences and Hospital, Jammu, India, over a period of one year and six months.

A total of 126 participants aged 30 years and above with established T2DM, diagnosed according to the American Diabetes Association (ADA) guidelines, were selected through simple random sampling from outpatients visiting internal medicine and nephrology clinics for ongoing care or monitoring. All data analyses were performed retrospectively.

Sample size calculation for the comparison of two independent means was done using the following formula:

$n = 2 (Z_\alpha/2 + Z_\beta)^2 / d^2$ [15].

Where *n* = sample size required per group, *Zα/2* = Z value corresponding to the desired level of significance (α = 0.05), *Zβ* = Z value corresponding to the desired power (80% power), and *d* = effect size (Cohen’s d). Thus, n = 2 (1.96 + 0.84)² / (0.5)² = 60.32, which corresponds to ≈ 60 participants per group. The required sample size per group was 60. The final sample size taken for our study is 126 participants (with albuminuria = 66; without albuminuria = 60).

Exclusion criteria included type 1 diabetes, active urinary or respiratory infections, hepatic or renal disorders, malignancy, hypertension, hematological diseases, nephrotic syndrome, renal artery stenosis, or reduced eGFR without microalbuminuria. Patients on exogenous steroids or sex hormones were also excluded.

Data collection

Prior to participation, the research methodology was thoroughly explained to each participant, with written informed consent secured from all individuals. Information was systematically gathered through a standardized data collection form encompassing participant demographics (age and gender) alongside clinical characteristics, including diabetes duration, anthropometric measurements (height and weight), and calculated BMI.

Laboratory assessment and clinical parameters

Biochemical evaluations included fasting blood sugar (FBS), glycated hemoglobin (HbA1c), kidney function markers, including blood urea and serum creatinine, and inflammatory biomarkers, including C-reactive protein (CRP), IL-6, total white cell count (TLC), and differential neutrophil and lymphocyte counts. The NLR was subsequently calculated from these values.

Markers of early diabetic nephropathy and classification

Early DN was assessed using standardized diagnostic markers. Urinary albumin excretion (UAE) was measured as the cornerstone for detecting incipient DN, with microalbuminuria recognized as the earliest clinical manifestation. Spot urine samples were analyzed for urinary albumin-to-creatinine ratio (UACR), which corrects for variations in urine concentration and provides a reliable measure of albuminuria. Estimated glomerular filtration rate (eGFR), calculated using the Chronic Kidney Disease Epidemiology Collaboration (CKD-EPI) [16] equation, was employed to evaluate renal function and detect subtle declines in filtration capacity before overt impairment. For the purpose of this study, early DN was defined as persistent microalbuminuria (UACR = 30-300 mg/g, confirmed on at least two occasions three to six months apart) with preserved renal function (eGFR ≥60 mL/min/1.73 m²; G1-G2).This definition is consistent with the Kidney Disease: Improving Global Outcomes (KDIGO) 2022 guidelines and the ADA-KDIGO consensus report [17,18].

Statistical analysis

Statistical analysis was done using IBM SPSS version 21 (IBM Corp., Armonk, NY). Descriptive data were expressed in terms of percentages and were compared using the chi-square test. Continuous data were expressed in terms of mean and standard deviation and compared using Student's t-test. Pearson's correlation coefficient was used to find the relationship between NLR and clinical parameters. A p-value less than 0.05 was considered statistically significant; otherwise, it was considered non-significant.

## Results

A total of 126 diagnosed diabetic patients were included in the study. Of these, 66 patients had DN and 60 had normal UAE. The two groups were compared for various variables such as demographic, laboratory, and renal function parameters. Correlation between NLR and various renal function parameters and inflammatory markers was also studied.

Table 1 presents comparisons between groups for variables including age, gender, BMI, FBS, and HbA1c. Patients showed similar age distribution and BMI in kg/m2 (p = 0.306 and 0.271, respectively) with no gender distribution differences (p = 0.363). However, FBS and HbA1c were significantly elevated in patients with albuminuria.

**Table 1:** Demographic and laboratory parameters of patients with type 2 diabetes mellitus. * Indicates statistical significance. Continuous variables were analyzed using Student's t-test. Descriptive data were compared using the chi-square test. FBS: fasting blood sugar; HbA1c: glycated hemoglobin.

Variables	Patients without albuminuria (n = 60)	Patients with albuminuria (n = 66)	Test statistics (t/χ²)	p-value
Age in years (Mean ± SD)	53.98 ± 13.07	56.23 ± 11.51	t = −1.03	0.306
Gender	M (39)	M (37)	χ² = 0.71	0.363
	F (21)	F (29)		
Height in meters (Mean ± SD)	1.58 ± 0.17	1.62 ± 0.12	t = −1.54	0.127
Weight in kg (Mean ± SD)	63.09 ± 6.49	65.25 ± 9.93	t = −1.43	0.155
BMI in kg/m^2 ^(Mean ± SD)	25.96 ± 4.54	26.90 ± 4.97	t = −1.10	0.271
FBS in mg/dL (Mean ± SD)	147.19 ± 34.28	174.46 ± 43.72	t = −3.87	0.002*
HbA1c in mmol/mol (Mean ± SD)	52.6 ± 17.4	59.7 ± 19.8	t = 9.46	0.034*

Table 2 shows the comparison of hematological parameters between the two groups. The NLR was significantly elevated in patients with albuminuria, with statistical significance (p < 0.001). Conversely, individual values, including TLC, differential neutrophil, and lymphocyte counts, showed no significant differences between the groups.

**Table 2:** Comparison of hematological parameters associated with early diabetic nephropathy in T2DM patients with or without albuminuria. * Indicates statistical significance. Continuous variables were compared by using the Student's t-test. T2DM: type 2 diabetes mellitus; TLC: total leukocyte count; NLR: neutrophil-to-lymphocyte ratio.

Variables	Without albuminuria (n = 60)	With albuminuria (n = 66)	t-test	p-value
TLC/mm^3 ^(Mean ± SD)	7428 ± 1664.86	7896 ± 2164.32	t = −1.37	0.179
Neutrophil/mm^3 ^(Mean ± SD)	5601.75 ± 1066.17	5995.91 ± 1164.32	t = −1.98	0.052
Lymphocyte/mm^3 ^(Mean ± SD)	2498.97 ± 834.92	2349.38 ± 762.12	t = −1.05	0.296
NLR (Mean ± ​​​​​​​SD)	1.96 ± 0.54	3.06 ± 0.98	t = −7.90	0.001*

Table 3 compares renal parameters between groups. eGFR was calculated using the CKD-EPI formula. Patients with albuminuria demonstrated significantly lower eGFR in ml/min/1.73 m^2^ versus those without albuminuria, with statistical significance (p = 0.001). Serum creatinine levels were elevated in the albuminuria group. Blood urea and blood urea nitrogen (BUN, in mg/dL) levels showed no significant differences between groups.

**Table 3:** Comparison of renal parameters associated with early diabetic nephropathy in T2DM patients with or without albuminuria. * Indicates statistical significance. Continuous variables were compared using Student's t-test. T2DM: type 2 diabetes mellitus; eGFR: estimated glomerular filtration rate.

Renal function test	Without albuminuria (n = 60) (Mean ± SD)	With albuminuria (n = 66) (Mean ± SD)	t-test	p-value
Blood urea in mg/dL (Mean ± SD)	28.79 ± 14.18	32.96 ± 18.91	t=−1.3	0.167
Serum creatinine in mg/dL (Mean ± SD)	0.78 ± 0.13	1.09 ± 0.19	t=−10.2	0.001*
Blood urea nitrogen in mg/dL (Mean ± SD)	11.90 ± 6.10	13.46 ± 6.21	t=−1.4	0.158
eGFR in mL/min/1.73 m^2 ^(Mean ± SD)	103.35 ± 9.26	82.41 ± 8.94	t=12.8	0.001*

**Figure 1:**
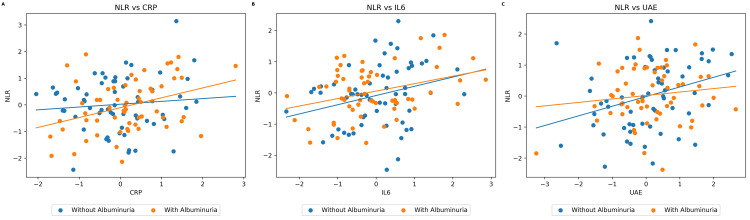
Correlation between NLR and CRP, IL6, and UAE by albuminuria status. Pearson correlation coefficients (r) are used to calculate the coefficient of correlation between each marker. NLR: neutrophil-to-lymphocyte ratio; CRP: C-reactive protein; IL6: interleukin 6; UAE: urinary albumin excretion.

Figure 1 shows the correlation analysis demonstrating that NLR showed a significant positive association with CRP (r = 0.193, p = 0.044) and IL-6 (r = 0.255, p = 0.007). However, no significant correlation was observed between NLR and UAE (r = 0.090, p = 0.654). These findings suggest that elevated NLR is significantly associated with increased albuminuria, higher inflammatory markers, and reduced renal function.

**Figure 2:**
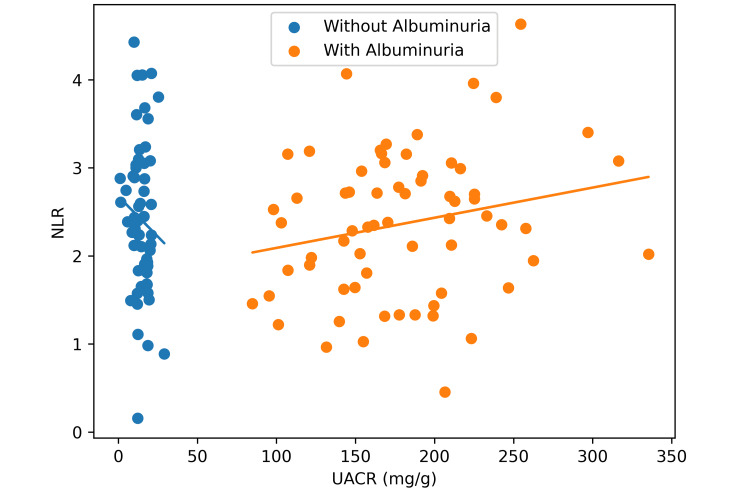
Correlation between NLR and UACR by albuminuria status. NLR: neutrophil-to-lymphocyte ratio; UACR: urinary albumin-to-creatinine ratio.

Figure 2 is a scatter plot showing the correlation between NLR and UACR based on albuminuria status (r = 0.301, p < 0.001), with patients having albuminuria demonstrating a positive correlation, whereas patients without albuminuria exhibited a tight clustering of data points at low UACR levels, displaying no apparent linear relationship with NLR.

**Figure 3:**
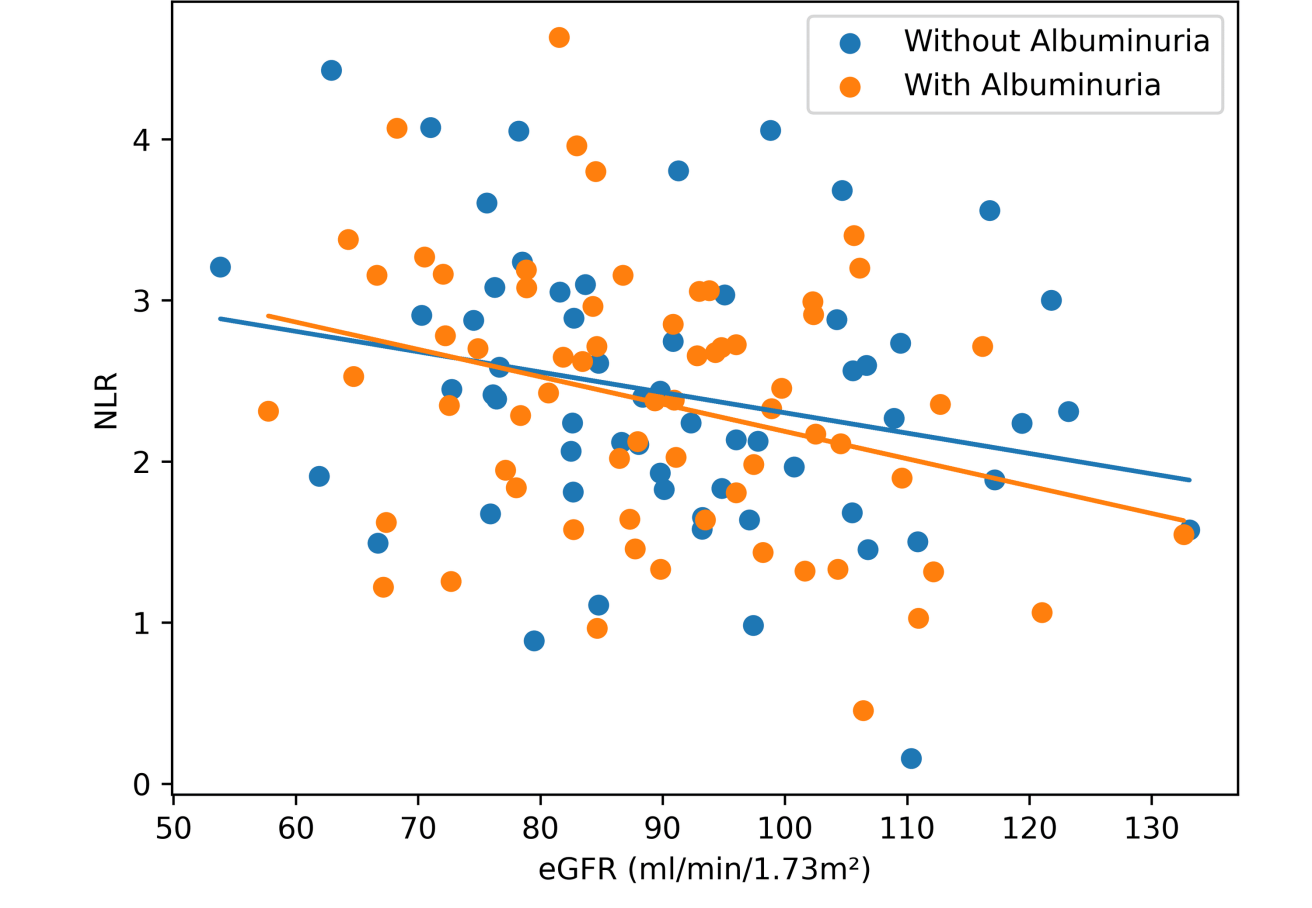
Correlation between NLR and eGFR by albuminuria status. NLR: neutrophil-to-lymphocyte ratio; eGFR: estimated glomerular filtration rate.

Figure 3 shows a scatter plot examining the relationship between NLR and eGFR (r = -0.228, p = 0.032) indicating inverse correlation as both groups (with and without albuminuria) show downward-sloping regression lines, but when correlated with an increasing NLR (heightened systemic inflammation), the group with albuminuria exhibited a slightly steeper regression slope, potentially indicating a more pronounced inflammatory response to declining kidney function in this subset of cohort.

## Discussion

DN is one of the most prevalent microvascular complications among individuals with T2DM, affecting approximately one-fifth to two-fifths of the diabetic population. This condition has emerged as the primary driver of ESRD worldwide [19]. While glomerular damage is considered an early sign of DN, microalbuminuria is a strong indicator of DN progression [20]. The progression of DN is significantly driven by the accumulation of advanced glycation end-products (AGEs). These compounds promote renal damage primarily by inducing oxidative stress and upregulating the production of various growth factors and pro-inflammatory mediators. A central mechanism occurs upon binding of AGEs to their specific receptor (RAGE), which activates key intracellular signaling pathways, including mitogen-activated protein kinase/extracellular signal-regulated kinase (MAPK/ERK), JNK, nuclear factor-kappa B (NF-κB), and tumor necrosis factor-beta (TGF-β). This cascade amplifies cellular oxidative stress and inflammatory responses, alters gene expression, and is fundamentally linked to the pathogenesis and advancement of T2DM and its microvascular complications [21]. Research has demonstrated that inflammatory mediators are central to T2DM pathogenesis and its microvascular sequelae. Renal inflammation is especially significant in driving DN progression [22]. Growing focus on early diabetic complication screening has led to increased use of the NLR as an inflammatory marker reflecting immune balance. Elevated NLR correlates with poor glycemic control and serves as a diabetic monitoring tool [23]. This study’s finding of elevated neutrophils and reduced lymphocytes in T2DM patients aligns with established literature reflecting the chronic inflammatory state of T2DM, where neutrophilia serves as a marker of systemic inflammation signifying active immune response, and the accompanying lymphopenia, a recognized feature of T2DM complications, is attributed to oxidative DNA damage and apoptosis of peripheral blood lymphocytes in peripheral circulation [24]. This study found higher TLC in T2DM patients with albuminuria versus those without, but the difference was not statistically significant. However, NLR was significantly elevated in T2DM patients with albuminuria compared to those without albuminuria (P < 0.001). Our results are consistent with Huang et al. [25], who reported significantly elevated NLR in T2DM patients with DN compared to T2DM without nephropathy. Similarly, Azab et al. [26], in a three-year follow-up study, identified NLR as a prognostic marker for declining renal function in T2DM patients. Chittawar et al. [27] similarly observed elevated NLR levels in DN patients compared to those without. Furthermore, Gurmu et al. [28] reported higher NLR values in the DN group versus those without DN in T2DM patients. On comparing renal function parameters in the present study, in patients with albuminuria, serum creatinine level was found to be significantly higher, and eGFR was found to be significantly lower. Gupta et al. [29] reported similar findings, observing significant increases in serum creatinine levels with progressive albuminuria. In our study, correlation analysis revealed a significant positive relationship between NLR and UACR, CRP, and IL-6. In contrast, NLR was negatively correlated with eGFR. Similar positive associations between NLR and inflammatory markers such as IL-6 and CRP have been reported by Karava et al. [30]. Consistent with our findings, Okyay et al. [31] also reported a positive correlation of NLR with IL-6 and CRP.

Limitations

This study's limitations include its small sample size, which may limit the generalizability of the results. Further research with a prospective design and repeated NLR measurements will shed more light on the role of NLR as a marker of inflammation and its potential as a risk factor for DN.

Future research and directions

Although NLR shows potential as a new tool for early diagnosis of nephropathy in T2DM, a prospective clinical study on a large number of patients is warranted to establish the effect.

## Conclusions

This study identified NLR as a cost-effective and readily accessible inflammatory biomarker that is associated with albuminuria in patients with T2DM. The findings suggest that NLR, derived from routine differential blood counts, holds particular promise for the early identification of DN in resource-constrained clinical settings where specialized inflammatory assays may be unavailable. The significant correlations observed between NLR and established renal impairment markers (UACR and eGFR), as well as with inflammatory cytokines (IL-6 and CRP), reinforce the role of systemic inflammation in the pathogenesis of ESRD. These results align with the growing recognition of inflammatory pathways in the development of microvascular complications. Consequently, NLR may serve as a practical tool for stratifying the risk of incipient DN.

## References

[1] Heald AH, Stedman M, Davies M (2020). Estimating life years lost to diabetes: outcomes from analysis of National Diabetes Audit and Office of National Statistics data. Cardiovasc Endocrinol Metab.

[2] Cole JB, Florez JC (2020). Genetics of diabetes mellitus and diabetes complications. Nat Rev Nephrol.

[3] Sun H, Saeedi P, Karuranga S (2022). IDF Diabetes Atlas: global, regional and country-level diabetes prevalence estimates for 2021 and projections for 2045. Diabetes Res Clin Pract.

[4] Rout P, Jialal I (2026). Diabetic nephropathy. StatPearls.

[5] Johns T, Jaar BG (2013). U.S. Centers for Disease Control and Prevention launches new chronic kidney disease surveillance system website. BMC Nephrol.

[6] Tsalamandris S, Antonopoulos AS, Oikonomou E (2019). The role of inflammation in diabetes: current concepts and future perspectives. Eur Cardiol.

[7] Subramani M, Anbarasan M, Shanmugam D, Muthumani LN, Vasudevan P (2023). Role of neutrophil-lymphocyte ratio as a prognostic marker for type 2 diabetic nephropathy among Indians. Bioinformation.

[8] Shiny A, Bibin YS, Shanthirani CS (2014). Association of neutrophil-lymphocyte ratio with glucose intolerance: an indicator of systemic inflammation in patients with type 2 diabetes. Diabetes Technol Ther.

[9] Abdel-Moneim A, Semmler M, Abdel-Reheim ES, Zanaty MI, Addaleel W (2019). Association of glycemic status and interferon-γ production with leukocytes and platelet indices alterations in type 2 diabetes. Diabetes Metab Syndr.

[10] Kocak MZ, Aktas G, Erkus E, Yis OM, Duman TT, Atak BM, Savli H (2019). Neuregulin-4 is associated with plasma glucose and increased risk of type 2 diabetes mellitus. Swiss Med Wkly.

[11] Shoelson SE, Lee J, Goldfine AB (2006). Inflammation and insulin resistance. J Clin Invest.

[12] Pickup JC (2004). Inflammation and activated innate immunity in the pathogenesis of type 2 diabetes. Diabetes Care.

[13] Tanaka T, Itoh H, Doi K (1999). Down regulation of peroxisome proliferator-activated receptorgamma expression by inflammatory cytokines and its reversal by thiazolidinediones. Diabetologia.

[14] American Diabetes Association (2019). Glycemic targets: standards of medical care in diabetes-2019. Diabetes Care.

[15] American Diabetes Association (2004). Nephropathy in diabetes. Diabetes Care.

[16] Miller WG, Kaufman HW, Levey AS (2022). National Kidney Foundation Laboratory Engagement Working Group recommendations for implementing the CKD-EPI 2021 race-free equations for estimated glomerular filtration rate: practical guidance for clinical laboratories. Clin Chem.

[17] Kidney Disease: Improving Global Outcomes (KDIGO) Diabetes Work Group (2022). KDIGO 2022 clinical practice guideline for diabetes management in chronic kidney disease. Kidney Int.

[18] de Boer IH, Khunti K, Sadusky T (2022). Diabetes management in chronic kidney disease: a consensus report by the American Diabetes Association (ADA) and Kidney Disease: Improving Global Outcomes (KDIGO). Diabetes Care.

[19] Sagoo MK, Gnudi L (2020). Diabetic nephropathy: an overview. Methods Mol Biol.

[20] Retnakaran R, Cull CA, Thorne KI, Adler AI, Holman RR (2006). Risk factors for renal dysfunction in type 2 diabetes: U.K. Prospective Diabetes Study 74. Diabetes.

[21] Khalid M, Petroianu G, Adem A (2022). Advanced glycation end products and diabetes mellitus: mechanisms and perspectives. Biomolecules.

[22] Lim AK, Tesch GH (2012). Inflammation in diabetic nephropathy. Mediators Inflamm.

[23] Khandare SA, Chittawar S, Nahar N, Dubey TN, Qureshi Z (2017). Study of neutrophil-lymphocyte ratio as novel marker for diabetic nephropathy in type 2 diabetes. Indian J Endocrinol Metab.

[24] Abiri E, Mirzaii M, Moghbeli M, Atashi A, Harati AA (2024). Investigating the relationship between lymphocyte cells apoptosis and DNA damage and oxidative stress and therapeutic and clinical outcomes of COVID-19 elderly patients. BMC Infect Dis.

[25] Huang W, Huang J, Liu Q, Lin F, He Z, Zeng Z, He L (2015). Neutrophil-lymphocyte ratio is a reliable predictive marker for early-stage diabetic nephropathy. Clin Endocrinol (Oxf).

[26] Azab B, Daoud J, Naeem FB (2012). Neutrophil-to-lymphocyte ratio as a predictor of worsening renal function in diabetic patients (3-year follow-up study). Ren Fail.

[27] Chittawar S, Dutta D, Qureshi Z, Surana V, Khandare S, Dubey TN (2017). Neutrophil-lymphocyte ratio is a novel reliable predictor of nephropathy, retinopathy, and coronary artery disease in Indians with type-2 diabetes. Indian J Endocrinol Metab.

[28] Gurmu MZ, Genet S, Gizaw ST, Feyisa TO, Gnanasekaran N (2022). Neutrophil-lymphocyte ratio as an inflammatory biomarker of diabetic nephropathy among type 2 diabetes mellitus patients: a comparative cross-sectional study. SAGE Open Med.

[29] Gupta N, Karoli R, Singh PS, Shrivastava A (2026). The relationship between neutrophil/lymphocyte ratio, albuminuria and renal dysfunction in diabetic nephropathy. J Indian Acad Clin Med.

[30] Karava V, Kondou A, Dotis J, Taparkou A, Farmaki E, Kollios K, Printza N (2024). Exploring systemic inflammation in children with chronic kidney disease: correlates of interleukin 6. Pediatr Nephrol.

[31] Okyay GU, Inal S, Oneç K (2013). Neutrophil to lymphocyte ratio in evaluation of inflammation in patients with chronic kidney disease. Ren Fail.

